# Supplementary material for: Complexome profiling of the Chlamydomonas psb28 mutant reveals TEF5 as an early PSII assembly factor
Source: Plant Cell. 2025 Mar 17;37(6):koaf055. doi: 10.1093/plcell/koaf055 (PMC12164590; doi:10.1093/plcell/koaf055)
Supplement: koaf055_Supplementary_Data [file koaf055_supplementary_data.zip › psb28_interactive.zip!/plots/intensity/qualityPlots/peptides/GAP3_Cre01.g010900.t1.2.html]

### Cre01.g010900.t1.2

Trivial name: GAP3  
SeqCoverage [median]: 29.90  
Mass: 40.30 kDa  
  
 

| Sequence | MissedCleavage | Mass [Da] | wtIntensity | muIntensity | Score |
| --- | --- | --- | --- | --- | --- |
| AAALNIVPTTTGAAK | 0 | 1397.79 | 4943.13 | 17950.38 | 129.70 |
| AVSLVLPSLK | 0 | 1025.65 | 1142.14 | 2426.45 | 71.50 |
| DKDIPTFVVGVNEGDYK | 1 | 1894.93 | 0.00 | 720.40 | 31.46 |
| GTMTTTHSYTGDQR | 0 | 1554.67 | 1196.37 | 3020.80 | 91.21 |
| IVDDSHISVDGK | 0 | 1283.64 | 3677.57 | 13192.72 | 123.95 |
| KTFAEEVNAAFR | 1 | 1381.70 | 0.00 | 477.59 | 58.17 |
| TFAEEVNAAFR | 0 | 1253.60 | 572.84 | 3324.97 | 133.13 |
| VLITAPAK | 0 | 811.52 | 0.00 | 2755.35 | 83.61 |
| VVDLAEVTAK | 0 | 1043.59 | 939.06 | 5345.63 | 99.80 |
| VVDLAEVTAKK | 1 | 1171.68 | 0.00 | 1175.17 | 58.17 |
| YDSTLGTFAADVK | 0 | 1386.67 | 0.00 | 1653.99 | 113.47 |
|  |  |  | 12471.1 | 52043.4 |  |
